# Effects of silver nanoparticle coating on peri-implant mucosa and microbiome

**DOI:** 10.1186/s40729-025-00664-0

**Published:** 2025-12-27

**Authors:** Ryutaro Ito, Yusuke Taniguchi, Tadahiro Kashiwamura, Hirofumi Kido, Kae Kakura, Nao Suzuki

**Affiliations:** 1 Section of Oral Implantology, Department of Oral Rehabilitation, Fukuoka Dental College, Fukuoka, Japan; 2 Department of Preventive and Public Health Dentistry, Fukuoka Dental College, Fukuoka, Japan; 3 Oral Medicine Research Center, Fukuoka Dental College, Fukuoka, Japan

**Keywords:** Silver nanoparticle, Peri-implantitis, Peri-implant mucositis, Microbiome, Odor measurement

## Abstract

**Aim:**

We conducted a study to investigate whether a silver nanoparticle (AgNP) coating on the surface of an implant superstructure could alter the microbiome of peri-implant tissues and to determine whether the AgNP coating would result in an improvement of gingival conditions and be effective in suppressing malodors.

**Method:**

We conducted a single-blind, parallel group comparative study in 19 patients undergoing implant maintenance. The 9 patients in the experimental group were treated by applying an AgNP coating after ultrasonic cleaning of the implant superstructure. Ultrasonic cleaning alone was performed on the 10 patients in the control group. The efficacy of the AgNP coating was evaluated by the following procedures conducted at baseline and after 3 months: measuring the modified gingival index (mGI), analyzing odor patterns through organoleptic test and olfactometric device readings of the implant superstructure, and determining the composition of the peri-implant microbiome.Registry: the Ethics Committee for Clinical Research of Fukuoka Gakuen, TRN: 530, Registration date: 30 March 2022.

**Results:**

The mGI values in the intervention group were significantly decreased (*p* = 0.043) than in the control group. In the organoleptic test, no significant intergroup differences were found in the sensory scores, but the sensory comments indicated that the odor type had changed in the experimental group. Principal component analysis (PCA) of the odor patterns at baseline and after 3 months revealed a change in the axis of the first principal component in the experimental group, but no change in the control group. A comparison of the peri-implant microbiome composition between the experimental group and the control group after three months revealed that the experimental group exhibited a significantly higher relative abundance of *Neisseria oralis* and *Ottowia* species, and a significantly lower relative abundance of *Veillonella parvula*, *Fretibacterium fastidiosum*, and *Tannerella forsythia* than the control group.

**Conclusion:**

These findings suggest that the AgNP coating of the implant superstructure changed the composition of the microbiome, and that such a change may improve gingival conditions and provide a deodorizing effect.

## Introduction

Dental implant therapy is currently widely performed as one form of prosthodontic treatment, but implant therapy can cause peri-implantitis when a microbial infection occurs. Treatment of peri-implantitis is broadly classified into surgical and nonsurgical approaches [1, 2]. Examples of nonsurgical approaches include topical application of chlorhexidine gluconate or a tetracycline antibiotic, oral administration of antimicrobial drugs, etc. Surgical treatments include scaling, air abrasion, and Er: YAG laser therapy [3]. These therapeutic modalities have not yielded clinically effective outcomes, so the emphasis has shifted to disease prevention [4].

The primary cause of peri-implantitis is attributed to specific bacteria in the peri-implant environment, including periodontal pathogens [5–7]. These anaerobic bacteria are known to release malodorous compounds, and also contribute to the particularly offensive odors associated with peri-implantitis [8, 9]. On the other hand, malodorous exudate may be noticed upon removal of the implant superstructure, regardless of the presence or absence of peri-implantitis. Since unpleasant malodors in the oral cavity are believed to be associated with dysbiosis of the microbiome, peri-implant tissues that exhibit an unpleasant malodor but are clinically diagnosed as healthy may have a high risk of peri-implantitis.

Therefore, control of the peri-implant microbiome is crucial for preventing peri-implantitis [4]. Commonly reported measures for preventing peri-implantitis include periodic examinations, enhanced motivation for maintaining oral hygiene, oral hygiene instruction, professional care, continuous inoculation of the lactic acid bacterium *Lactobacillus salivarius* strain WB21 [10], and also incorporation of AgNPs into dental material [11].

Frequent use of antibiotics for dental treatment and the continuous use of oral care products containing antimicrobial or bactericidal agents contribute to the emergence of antimicrobial-resistant bacteria [12]. The World Health Organization has declared antimicrobial resistance a global health concern, and alternative approaches to maintain oral health including probiotics and AgNPs are receiving increased attention [13].

AgNPs are reported to exhibit strong antimicrobial properties [14–16]. Previous in vitro studies demonstrated that titanium discs coated with AgNPs provide antibacterial effects against bacteria associated with peri-implantitis. AgNP coating of implant abutments has also been shown to suppress accumulation of dental plaque [17], and coating of AgNPs on implant abutments has been confirmed to inhibit plaque adhesion [11]. Even so, the effects of AgNP coatings on the peri-implant microbiome in the oral cavity have remained unclear.

In this study, we tested whether coating the surface of implant superstructures with an AgNP aqueous solution (Pikash, Pikash Co., Kumamoto, Japan) improves the peri-implant microbiome through the antibacterial effects of AgNPs, and consequently enhances peri-implant soft tissue conditions and reduces malodor. This study was conducted as a single-blind, parallel-group comparative trial, in which participants were assigned to either aAgNP–coated group or a non-coated control group. Changes in the peri-implant microbiome, peri-implant mucosal condition, and malodor were evaluated using the modified Gingival Index (mGI), an organoleptic test, detection of malodor-causing molecules, and microbiome analysis.

## Materials and methods

### Study design

**Fig. 1:**
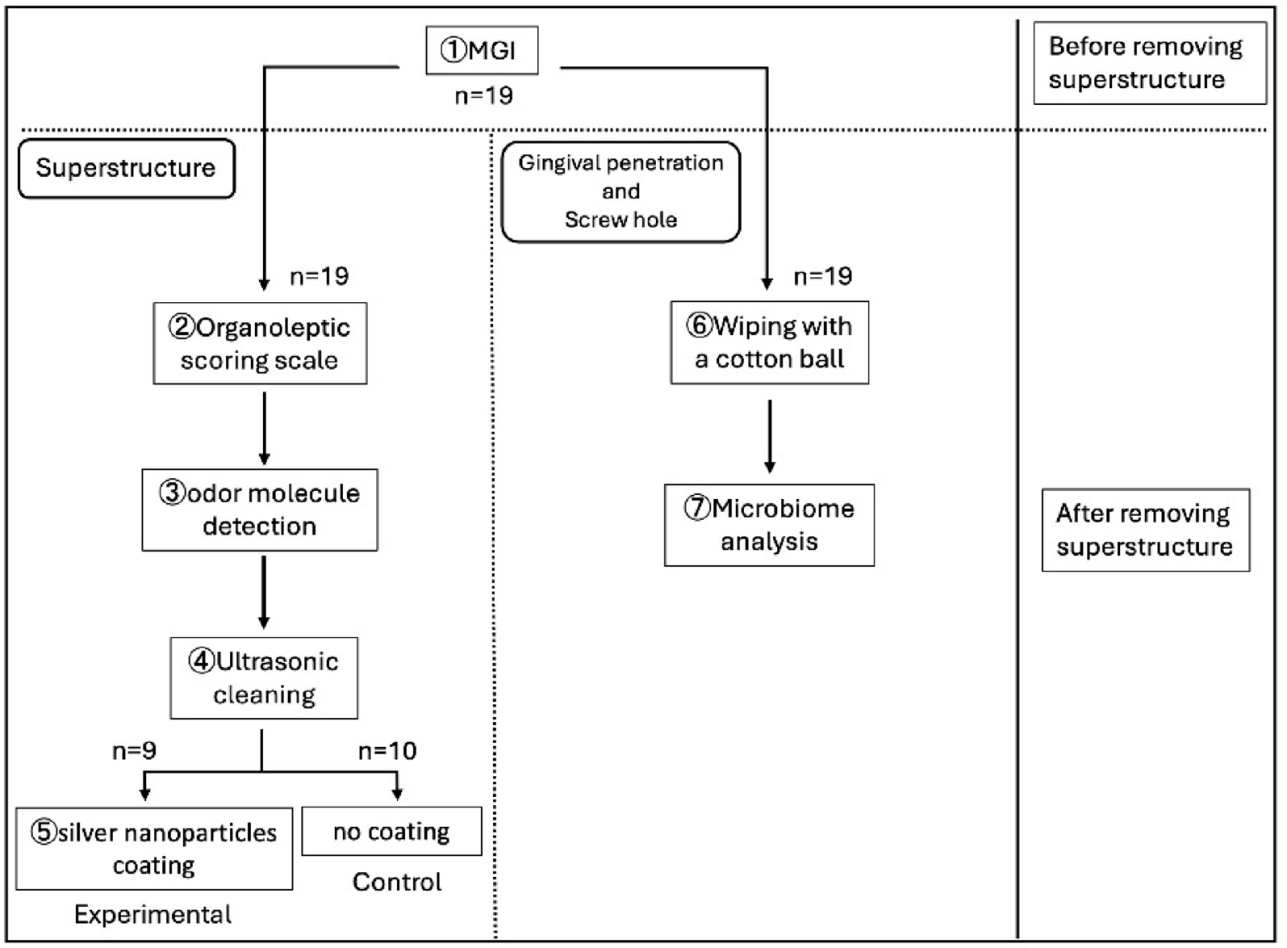
Flowchart of this study conducted as a single–blind parallel–group comparative trial: A second evaluation was conducted three months after the first evaluation

This study was conducted as a single-blind, parallel-group comparative study. Nineteen patients undergoing maintenance in our department (6 males and 13 females; mean age, 62.11 years) were numbered sequentially by order of entry. Inflammatory findings of peri-implant tissues were evaluated using mGI in nine subjects with even-numbers. Then the superstructure was removed, detection of odorants on the superstructure was performed using both a sensory evaluation and an olfactometric device (nose@MEMS, I-PEX Inc., Kyoto, Japan), ultrasonic cleaning was performed, and an AgNP coating was applied. Moreover, samples were collected from both the gingival permucosal region and the screw hole of the implant body (the inside of the implant body), and subjected to microbiome analysis. A second evaluation was conducted three months later, and the even-numbered patients were designated as the experimental group. Ten odd-numbered patients underwent the same procedures as the experimental group, except that the AgNP coating was not applied.The odd-numbered patients were designated as the control group (Fig. 1). All prosthetic superstructures in the 19 participants were zirconia crowns on titanium bases. Exclusion criteria were: (i) a history of silver allergy; (ii) poor systemic health; (iii) habitual smoking; (iv) psychiatric disorders that impair communication; (v) alcohol or drug dependence; (vi) age under 20 years; (vii) any other condition deemed inappropriate for study participation by the investigators; (viii) uncontrolled periodontal disease; (ix) severe occlusal dysfunction or marked bruxism; or (x) a history of implant treatment failure. None of the enrolled participants met any of these exclusion criteria.

The sensory examination and olfactometric odorant detection were also performed on an unseated superstructure (zirconia crown on titanium base) in one patient (hereinafter, the “unseated superstructure group”) to serve as reference data. The mGI was also measured in eight patients with peri-implantitis (hereinafter, the peri-implantitis group), and a sensory evaluation and olfactometric odorant detection were performed on the implants, including the superstructures after removal. The purpose, significance, method, and duration of the study were fully explained to the patients, and written consent to participate in the clinical trial was obtained from each patient with sufficient understanding. This study was conducted in accordance with the guidelines of the Declaration of Helsinki, approved by the Ethics Committee for Clinical Research of Fukuoka Gakuen (approval number 530, 30 March 2022), and registered with the University Hospital Medical Information Network (UMIN) Center (UMIN000042426).

### mGI (Modified gingival index)

During placement, the peri-implant examination areas around the superstructure were designated as six locations: the mesial papillary region, marginal region, and distal papillary region on both the buccal and lingual sides, and were evaluated on a 5-point scale from 0 to 4. The mean mGI score of these six sites was defined as the mGI score for each individual [10, 18] (Table 1). All examinations related to mGI were conducted by a single implant specialist who had received prior training in mGI evaluations.

**Table 1:** mGI score

0	Absence of inflammation
1	Mild inflammation; slight change in color, little change in color; little change in texture of any portion of the marginal or papillary gingival unit
2	Mild inflammation; criteria as above but involving the entire marginal or papillary gingival unit
3	Moderate inflammation; glazing, redness, edema, and / or hypertrophy of the marginal or papillary gingival unit
4	Severe inflammation; marked redness, edema and / or hypertrophy of the marginal or papillary gingival unit, spontaneous bleeding, congestion, or ulceration

### Organoleptic test for superstructure

The removed superstructure was placed in a 15 mL conical tube, and after closing the lid for 1 min to allow odor saturation, an organoleptic test was conducted at a distance of 1 cm from the conical tube. The test was conducted by four examiners, and assessment was performed on a 6-point scale from 0 to 5 based on the criteria following the method of Rosenberg et al. [19] The average value of the four scores was designated as the individual organoleptic score, and specific comments about the odors were also recorded (Table 2).

**Table 2:** Organoleptic test score

Category	Description
0: Absence of odor	Odor cannot be detected
1: Questionable odor	Odor is detectable, although the examiner could not recognize it as malodor
2: Slight malodor	Odor is deemed to exceed the threshold of malodor recognition
3: Moderate malodor	Malodor is definitely detected
4: Strong malodor	Strong malodor is detected, but can be tolerated by examiner
5: Severe malodor	Overwhelming malodor is detected and cannot be tolerated by examiner (examiner instinctively averts the nose)

### Detection of malodor-causing molecules

The odor analyzer comprised a petri dish, a pump, and a sensor connected by tubing. The removed implant superstructure was placed in a petri dish, the air in the petri dish was evacuated by the pump, and the evacuated air was then directed onto the sensor (nose@MEMS, I-PEX, Kyoto, Japan) for 30 s to obtain a quantitative odor measurement.

**Fig. 2:**
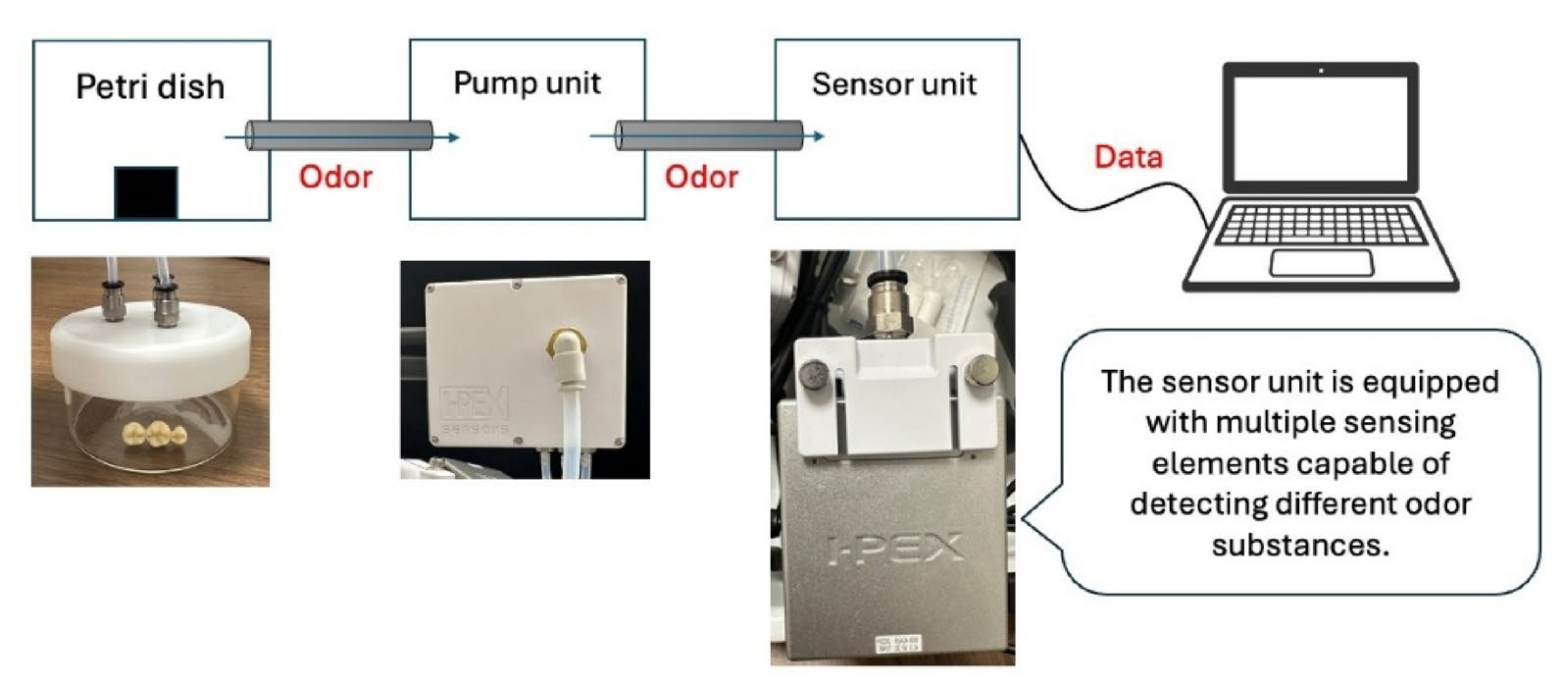
Olfactometric device used to detect odorants : The odor analyzer comprised a petri dish, a pump, and a sensor connected by tubing. The removed implant superstructure was placed in a petri dish, the air in the petri dish was evacuated by the pump, and the evacuated air was then directed onto the sensor for 30 seconds to obtain a quantitative odor measurement. Measurement was conducted three times for each patient with a 10-second interval between measurements. The odor sensor was connected to a personal computer by a USB cable to facilitate data acquisition

Measurement was conducted three times for each patient with a 10-second interval between measurements. PCA and dendrograms were used to analyze the odor data obtained from the olfactometric device. The odor sensor was connected to a personal computer by a USB cable to facilitate data acquisition (Fig. 2). The sensor itself is equipped with a detection element approximately 1.2 mm in diameter wherein many different sensing membranes have been coated onto a lead zirconate titanate (PZT) piezoelectric thin film and mounted on a sensor chip.

The measurement method involves applying voltage to the resonating sensing membranes, which allows odorants to adsorb onto the membranes, and calculating the quantity (odor intensity) based on the change in resonance frequency. The odor sensor can detect a variety of odorants and generate a response pattern by using sensitive membranes with different properties. Although the odor sensor can accommodate 180 types of sensing membranes, a preliminary experiment was conducted to select sensing membranes from the 180 types that would specifically react to the odor of peri-implantitis. The sensing membrane selection experiment was performed on the implant superstructures of four patients with suspected peri-implantitis.

Based on the measurement results, 15 types of sensitive membranes that exhibited different data with particularly strong responses were selected. The selected detection elements were 1026, 1029, 200 K, 200T, 200 V, 200 W, 200X, 200Y, 20CU, 20CV, 20CX, 20CY, 20CZ, 20DA, and 20DB.

### Ultrasonic cleaning

The superstructure was placed in a ziplock polyethylene bag (12 cm × 7 cm) containing 30 ml of sterile purified water (Nikko Pharmaceutical Co., Ltd., Japan), and ultrasonic cleaning was performed for 15 min using an ultrasonic cleaner (Ultrasonic cleaner, AU-12 C, Aiwa Medical Industry Co., Ltd., Tokyo).

### AgNP coating

**Fig. 3:**
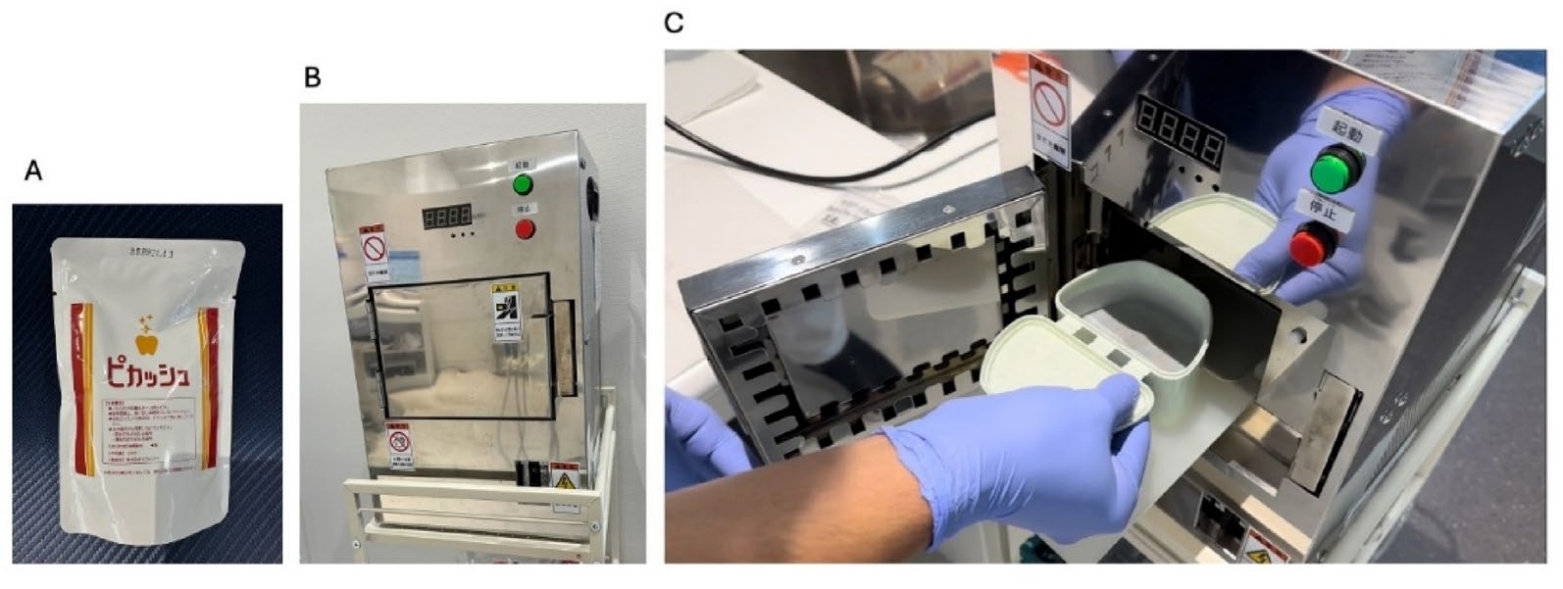
Silver nanoparticle coating using microwave irradiation device:** A** silver nanoparticle aqueous solution** B** microwave irradiator** C** Positioning of superstructure and case containing silver nanoparticle aqueous solution in microwave irradiator

A 150 mL aqueous solution of silver nanoparticles (Pikasshu, Pikasshu Co., Kumamoto, Japan) and the superstructure were placed in a plastic case, the case was installed in a microwave irradiator, and microwave irradiation was performed to coat the superstructure surface with AgNPs. The microwave irradiation device timer was set to 90 s and programmed to stop and begin cooldown when the temperature reached 55 ℃ (Fig. 3).

As a preliminary experiment, titanium plates (9 mm × 10 mm × 2 mm; KZR-CAD Ti Gr. 5, YAMAKIN Co., Ltd., Kochi, Japan) were coated or not with AgNP and rinsed five times with purified water. The coated and uncoated titanium plates were then examined using an ultra-high-resolution field-emission scanning electron microscope (SU8600), and elemental analysis including Energy Dispersive X-ray spectroscopy (EDX) was performed using AZtecLive/Ultim Max. Scanning electron microscopy (SEM) imaging confirmed the presence of dispersed nanoparticles exclusively on the AgNP-coated titanium plates. Elemental and EDX analyses also detected silver only on the coated plates (data not shown).

### Bacterial sampling

The permucosal penetration and the screw hole of the implant body (the inside of the implant body) were wiped with sterile cotton balls, and the cotton balls were then placed into microtubes containing 500 µL of PBS (Dulbecco’s Phosphate Buffered Saline, Lonza, USA) and mixed with a vortex mixer (Vortamix Mini Vortex Shaker, Cole-Parmer, USA) for 20 s. The cotton balls were removed, and the contents of the microtubes were cryopreserved at − 20 ℃.

### Microbiome analysis

Extraction of bacterial DNA was performed using a MORA-EXTRACT DNA extraction kit (Kyokuto Pharmaceutical Industrial Co., Ltd., Japan).

Microbiome analysis was performed using amplicon sequencing analysis (MiSeq, Illumina, USA) targeting the 16 S rDNA V3 to V4 (bacterial and archaeal) regions. Pro341F-Pro805R was used as the primer. QIIME1.8.0 8 usearch6.1.544_i86 was used to detect and remove chimeric sequences. Data analysis was performed using the RDP (Ribosomal database project) and the Microorganism Identification Database (TechnoSuruga Laboratory Co. Ltd., Japan). Based on the results obtained from sequence analysis, a comparative analysis of the relative abundance in the microbiome between the control group at the second measurement and the experimental group at the second measurement was performed using Linear discriminant analysis Effect Size (LEfSe) developed by Segata et al. [20].

### Statistical processing

The mGI scores and organoleptic test scores were compared using t-tests. When the odor threshold was set at 2 in the sensory test, the mean of the intra-class correlation measurements from the four evaluators was 0.856. PCA was used to evaluate the data obtained from odorant detection, and LEfSe was used for group comparison in the microbiome analysis.

## Results

### mGI

**Fig. 4:**
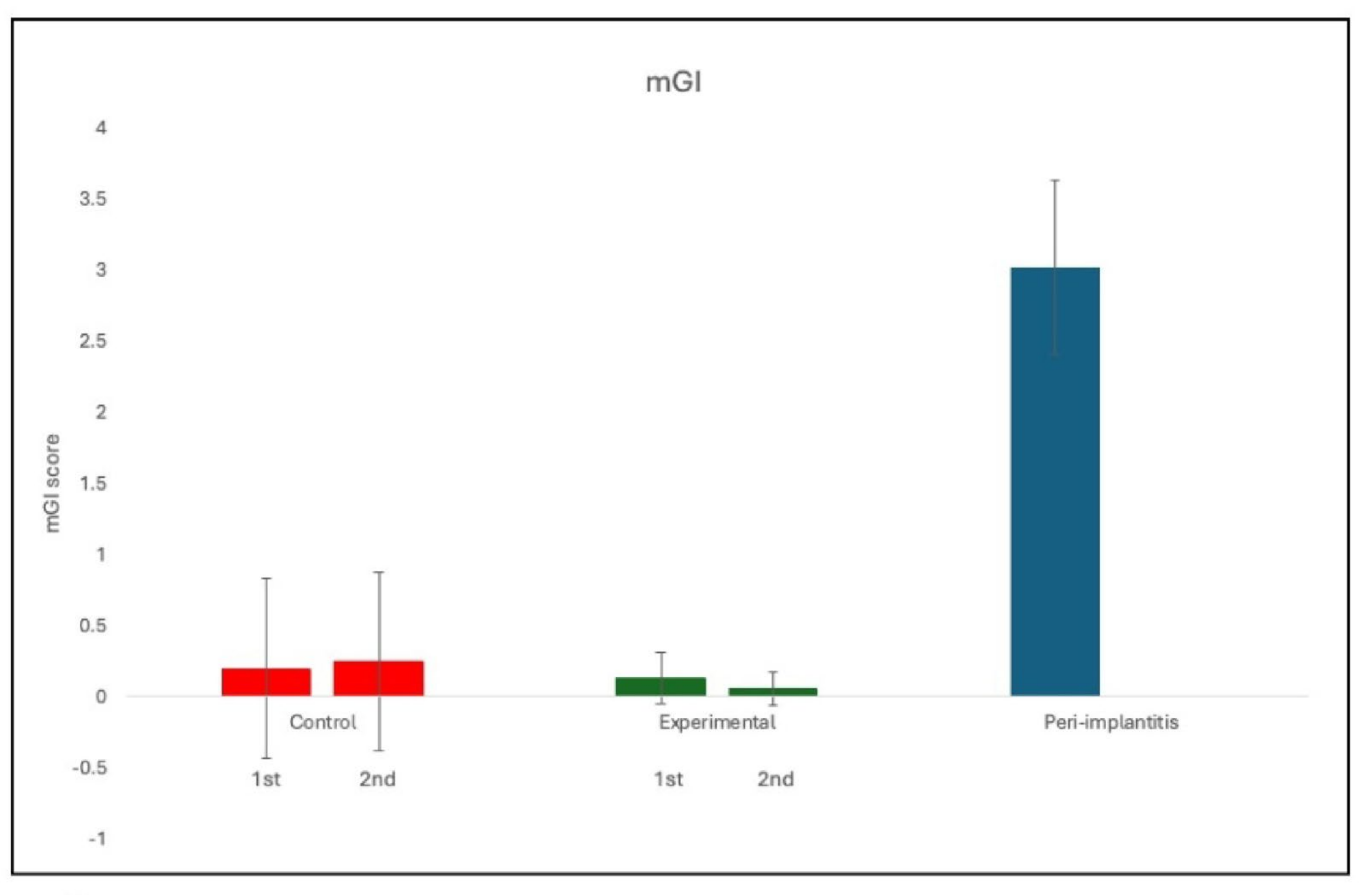
mGI score results: The respective mGI scores for the control group and experimental group were 0.2 and 0.13 at the first measurement and 0.25 and 0.06 at the second measurement. When comparing the magnitude of change between the two groups, the experimental group demonstrated a statistically significant decrease in mGI compared to the control group (*p* = 0.043). For comparison, the mGI score in the peri-implantitis group was 3.02 ± 0.61

The respective mGI scores for the control group and experimental group were 0.2 and 0.13 at the first measurement and 0.25 and 0.06 at the second measurement. When comparing the magnitude of change between the two groups, the experimental group demonstrated a statistically significant decrease in mGI compared to the control group (*P* = 0.043). For comparison, the mGI score in the peri-implantitis group was 3.02 ± 0.61 (Fig. 4).

### Organoleptic test

The respective organoleptic scores for the control group and experimental group were 2.76 ± 1.37 and 2.17 ± 1.16 in the first measurement, and 2.49 ± 1.22 and 1.86 ± 1.38 in the second measurement, and no significant differences between the first and second measurements were found in either group. Organoleptic test comments showed no change in the control group, which remained characterized by “fecal odor or rotten egg odor,” while the odor in the experimental group shifted from “rotten egg odor” to “fermentation odor.”

**Fig. 5:**
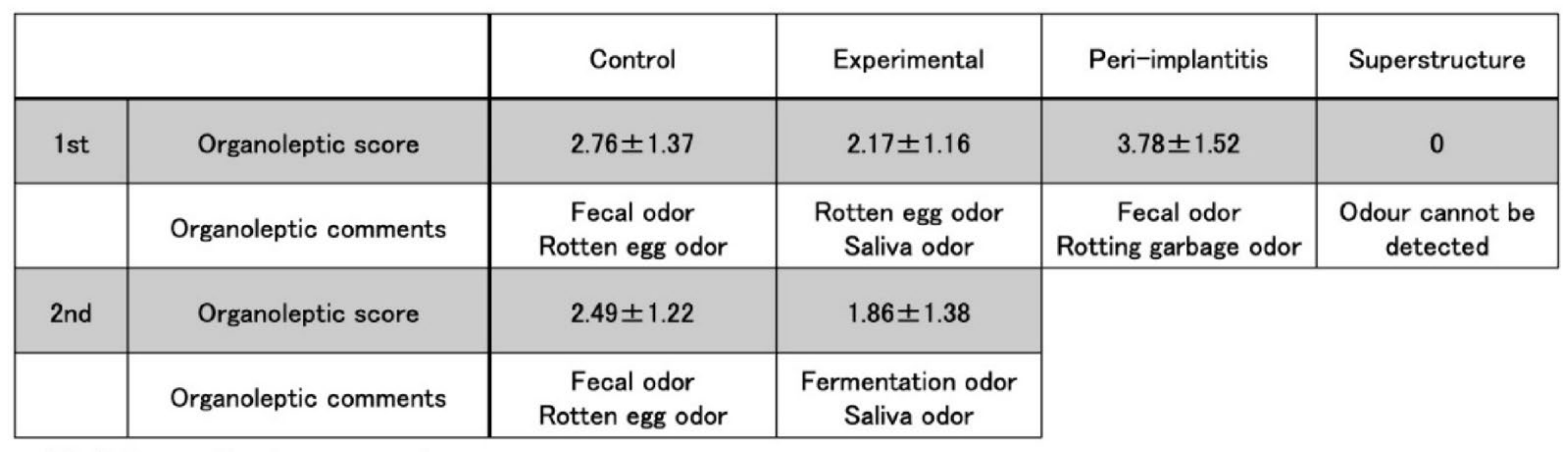
Organoleptic test results: No Significant differences were found in the respective organoleptic scores between first and second sessions in either groups. Organoleptic test comments showed no change in the control group, which remained characterized by "fecal odor or rotten egg odor ", while the odor in the experimental group shifted from "rotten egg odor " to fermentation odor"

Furthermore, in the peri-implantitis group, the organoleptic score was 3.78 ± 1.52 with comments of “fecal odor” or “rotting garbage odor,” while in the group with the unseated superstructure, the score was 0 with comments of “no odor detected above the odor threshold” (Fig. 5).

### PCA results for malodor detection

**Fig. 6:**
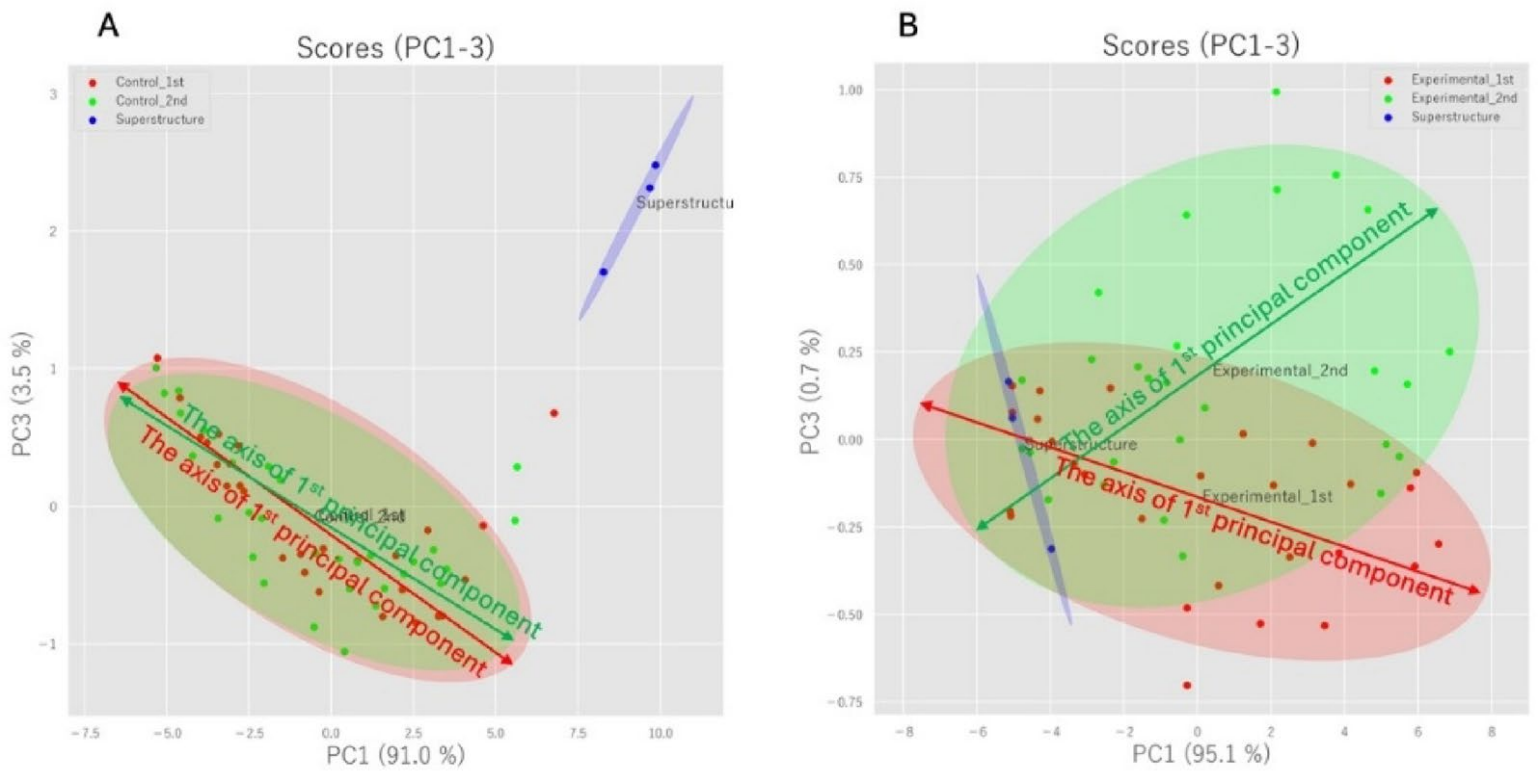
PCA results for odorant detection: there was no change in the axis of the first principal component between the first and second measurements in the control groups. However, there was a substantial change in the axis of the principal component between the first and second measurements in the experimental group.** A** PCA of the control group** B** PCA of the experimental group

In the intragroup comparison of the control group, the first principal component accounted for 91.0% of the total variance, and the second principal component accounted for 3.5%, and there was no change in the axis of the first principal component between the first and second measurements in the control group. In the intragroup comparison of the experimental group, however, the first principal component accounted for 95.1% of the total variance, and the second principal component accounted for 0.7%. Notably, in the experimental group there was a substantial change in the axis of the first principal component between the first and second measurements (Fig. 6).

### Results of microbiome analysis

**Fig. 7:**
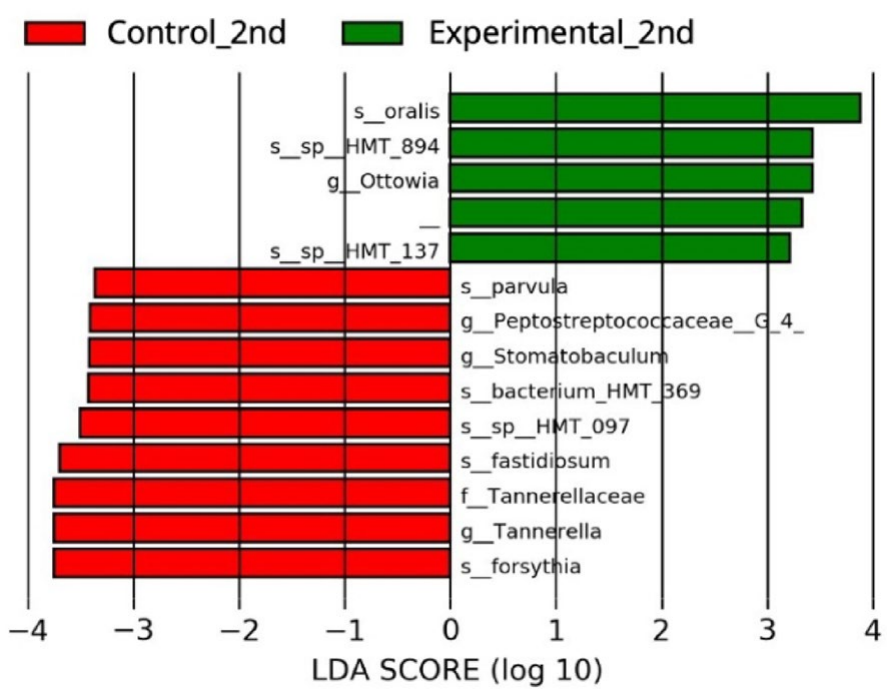
Result of microbiome analysis: When the microbiomes of the experimental group and control group in the second measurements were compared, the experimental group had significantly higher proportions of* Neisseria oralis* and* Ottowia *genus compared to the control group, and significantly lower proportions of* Veillonella parvula*,*Fretibacterium fastidiosum* and* Tannerella forsythia*

When the microbiomes of the experimental group and control group in the second measurement were compared, the experimental group had significantly higher proportions of *Neisseria oralis* and *Ottowia* genus compared to the control group, and significantly lower proportions of *Veillonella parvula*, *Fretibacterium fastidiosum*, and *Tannerella forsythia* (Fig. 7).

## Discussion

The aim of this study was to investigate whether coating the surface of implant superstructures with AgNPs that have been reported to exhibit antibacterial activity against peri-implantitis–associated pathogens [17] would modify the peri-implant microbiome, reduce the population of anaerobic bacteria responsible for peri-implantitis, improve the peri-implant mucosal condition, and consequently suppress oral malodor produced by anaerobic bacterial species. The mechanism of antimicrobial action involves AgNPs disrupting cellular membranes, affecting adenosine triphosphate production and DNA replication, altering gene expression, and oxidizing cellular biological compartments by generating reactive oxygen species (ROS). It has been reported that silver ions released by AgNPs in a biological environment can block the microbial respiratory chain at both the cytochrome oxidase and NADH-succinate dehydrogenase complexes [21]. Moreover, numerous reports indicate that incorporating AgNPs into dental material is effective in inhibiting the proliferation of intraoral bacteria [11, 14, 22–26]. Research utilizing the AgNP aqueous solution used in this study has also been conducted. In a report by Odatsu T et al. [11], the AgNP coating on denture bases inhibited the adhesion of oral *Candida* species to the denture bases and caused morphological deformations in vitro. In addition, Yoshiaki K et al. [26] have reported that titanium plates with AgNP coatings demonstrated substantial inhibition of *Staphylococcus aureus* colony-forming units in vitro, and that in a clinical trial, AgNP coatings on healing abutments resulted in a significant reduction of plaque adhesion.

In this study, mGI was used to confirm the presence or absence of inflammation and to verify that the subjects were evenly divided between the two groups regardless of the presence or absence of inflammation, since the inflammatory state of the gingiva may result in substantial individual variations in the peri-implant microbiome. The mGI serves as an index for assessing gingival health, and it records even minor localized inflammatory findings. Therefore, mGI is effective even in patients with minimal inflammation such as those in the present study [10, 27].

Based on the mGI results, both groups exhibited scores of 1 or lower, with only slight discoloration observed in the peri-implant gingiva, and no differences were detected between the two groups at baseline. This finding also corroborated that the study subjects had been randomly assigned. The pre- and post-intervention comparison showed that at the second measurement, the scores had increased in the control group, but the scores had decreased in the experimental group with the AgNP coating. A statistically significant difference in the amount of change was found between the two groups, suggesting that intervention with an AgNP coating in maintenance patients may contribute to maintaining peri-implant health. A previous study has reported that AgNP coatings exert an inhibitory effect on dental plaque adhesion [11]. In the present study, however, no visually detectable peri-implant plaque accumulation was found in the patients. Furthermore, quantitative analysis of total microbial counts in the swab samples collected from the peri-implant areas of the experimental group revealed no significant changes between the first and second measurements. These findings suggest that the AgNP coating may have affected the composition of the microbiome rather than the microorganism count per se.

The organoleptic test results revealed that the sensory scores in the control group were 2.76 ± 1.37 in the first measurement and 2.49 ± 1.22 in the second measurement, and no statistically significant difference was found. The organoleptic scores in the experimental group were 2.17 ± 1.16 in the first measurement and 1.86 ± 1.38 in the second measurement, and no statistically significant difference was found. The organoleptic comments for the control group in the first and second measurements remained unchanged as “fecal odor or rotten egg odor,” but the sensory comments in the experimental group changed from “rotten egg odor” in the first measurement to “fermentation odor” in the second measurement, suggesting that the AgNP coating may effectively alter the microbiome.

The “rotten egg odor” in halitosis originates in sulfur compounds that are produced by anaerobic bacteria of the genera *Fusobacterium*, *Prevotella*, *Porphyromonas*, and *Treponema* [28, 29]. These anaerobic bacteria are associated with dysbiosis in the oral cavity and are known to be key pathogens in periodontal disease and oral malodor. In contrast, the components of the “fermentation odor” in halitosis are acetic acid and lactic acid, which are produced by Gram-positive bacteria such as the species *Streptococcus*, *Lactobacillus*, and *Actinomyces* [30–32], which are considered to be involved in the stability of the oral microbiota. Therefore, our findings suggest that the AgNP coating may effectively alter the microbiome.

The olfactometric device utilized in the present study digitizes odorants and represents them using methods such as PCA. If the first principal component axis derived from PCA is the same, the odor types are identical, but a change in the axis reveals that the odor types are different. Based on the PCA results, while there was no change in the first principal component axis in the intragroup comparison of the control group, there was a change in the first principal component axis in the intragroup comparison of the experimental group, showing that the type of odor had changed. This finding corroborates the results of the sensory test.

Volatile sulfur compounds (VSCs) such as hydrogen sulfide, methyl mercaptan, and dimethyl sulfide are generally the main compounds in malodors originating in the mouth [8, 9]. In a preliminary study the VSCs of implants removed from peri-implantitis patients were measured by gas chromatography, but no VSCs were detected. The VSC concentration in the peri-implant microenvironment is considered to be quite low, and the microorganisms produce multiple metabolic products other than VSCs, including ammonia and unsaturated fatty acids, which makes the situation quite complex [33, 34]. Therefore, in this study, we utilized a highly sensitive olfactometric device, which is responsive to a variety of odiferous compounds. The fact that this device successfully measured peri-implant malodor in this study suggests that it can be applied to various implant-related studies in the future.

In this study the bacterial populations in the control and experimental groups were comprehensively analyzed using LEfSe. Based on the comparative analysis of bacterial flora between the experimental group and control group at 3 months post-intervention, the bacteria significantly more abundant in the experimental group were *Neisseria oralis* and *Ottowia* genus, while the bacteria significantly more abundant in the control group, in other words, the bacteria significantly less abundant in the experimental group, were *V.parvula*, *F.fastidiosum*, and *T.forsythia*. *N.oralis*, which was significantly more abundant in the experimental group, is a species of the genus *Neisseria*, which are known as a gram-negative, anaerobic, rod-shaped bacteria, and *N.oralis* is an indigenous microorganism in the oral cavity [35, 36]. It has been reported that bacteria of the genus *Ottowia* have been detected in trace amounts in human dental calculus, but the detailed characteristics of *Ottowia* remain largely unknown since it has only recently been identified [37, 38]. It has been reported that *V.parvula*, *F.fastidiosum*, and *T.forsythia*, which were significantly less abundant in the experimental group, are involved in peri-implantitis [39]. *V.parvula* is known as a commensal microorganism of the coating of the tongue and is recognized as a hydrogen sulfide-producing bacterium [40, 41]. *F.fastidiosum* is frequently detected in subgingival plaque from patients with chronic periodontitis and has been reported to produce acetic acid [42, 43]. *T.forsythia* is recognized as a member of the red complex that produces hydrogen sulfide and methyl mercaptan [44, 45]. These findings suggest that an AgNP coating may effect changes in the bacterial flora. Notably, the observed reduction in the proportion of bacterial species associated with peri-implantitis and periodontitis suggests a possible correlation with the improvement of the peri-implant gingival condition.

Based on these findings, this study suggests that an AgNP coating may alter the microbiome and concomitantly improve the gingival condition and suppress malodors. However, given the limited number of subjects (*n* = 19) and the fact that intervention timing coincided with quarterly maintenance visits, future studies with a larger subject population, a variety of intervention periods, and longer follow-up periods will be needed in the future. In addition, it is important to note that this study was conducted at a single center, which may limit the characteristics of the study population. Multicenter studies involving a broader range of patient backgrounds and clinical settings will be necessary to enhance the generalizability of the findings. The present study also did not evaluate the uniformity or durability of the silver nanoparticle coating on the titanium alloy surfaces. As such, additional investigations assessing surface characteristics of AgNP-coated materials are warranted.

## Conclusion

In this study, AgNP coating was suggested to reduce peri-implant pathogenic bacteria and modify the peri-implant microbiome. Furthermore, the results indicated potential improvements in gingival conditions and a suppressive effect on oral malodor. These findings suggest that the potential for suppressing oral malodor and preventing peri-implantitis in patients undergoing implant therapy.

## Data Availability

No datasets were generated or analysed during the current study.
